# Innovations in Providing HIV Index Testing Services: A Retrospective Evaluation of Partner Elicitation Models in Southern Nigeria

**DOI:** 10.9745/GHSP-D-24-00013

**Published:** 2024-10-29

**Authors:** Caesar C. Dibia, Pius Nwaokoro, Uduak Akpan, Otoyo Toyo, Simon Cartier, Olusola Sanwo, Ngozi Sydney-Agbor, Barinaada Afirima, Kunle Kakanfo, Uwem Essien, Christa Fischer Walker, Hadiza Khamofu, Satish Raj Pandey, Moses Bateganya

**Affiliations:** a Achieving Health Nigeria Initiative, Akwa Ibom, Nigeria.; b FHI 360, National Capital District, Papua New Guinea.; c Plateau Specialist Hospital, Jos, Nigeria.; d FHI 360, Abuja, Nigeria.; e Imo State University, Owerri, Nigeria.; f FHI 360, Lilongwe, Malawi.; g African Hub for Health and Innovation, Ottawa, Canada.; h Centre for Integrated Health Programs, Kaduna, Nigeria.; i FHI 360, Washington, DC, USA.

## Abstract

**Background:** This analysis aimed to evaluate the effectiveness of eliciting sexual partners from HIV-positive clients using the elicitation box model (where an HIV-positive index can report sexual contacts on paper and insert in a box for a health care provider to contact at a later time) compared to the conventional model (in which a health care provider elicits sexual contacts directly from clients) in Akwa Ibom, Southern Nigeria.

**Methods:** Between March 2021 and April 2022, data were collected from index testing registers at 4 health facilities with a high volume of HIV clients currently on treatment in 4 local government areas in Akwa Ibom State. Primary outcome analyzed was the elicitation ratio (number of partners elicited per HIV-index offered index testing services). Secondary outcomes were the index testing acceptance (index HIV-positive clients accepted index testing service), testing coverage (partners tested for HIV from a list of partners elicited from HIV-index accepted index testing services), testing yield (index partners identified HIV positive from index partners HIV-tested), and linkage rate (index partners identified HIV positive and linked to antiretroviral therapy).

**Results:** Of the total 2,705 index clients offered index testing services, 91.9% accepted, with 2,043 and 439 indexes opting for conventional elicitation and elicitation box models, respectively. A total of 3,796 sexual contacts were elicited: 2,546 using the conventional model (elicitation ratio=1:1) and 1,250 using the elicitation box model (elicitation ratio=1:3). Testing coverage was significantly higher in the conventional compared to the elicitation box model (*P*<.001). However, there was no significant difference in the testing yield (*P*=.81) and linkage rate using the conventional compared to elicitation box models (*P*=.13).

**Conclusion:** The implementation of the elicitation box model resulted in an increase in partner elicitation compared to the conventional model. Increasing the testing coverage by implementing the elicitation box model should be considered.

## Abstract (precis)

The implementation of the elicitation box model to elicit sexual partners of HIV-positive index cases resulted in an increase in partner elicitation compared to the conventional model.

## Teaser

**Key Findings:**

- Index testing is part of the strategies to identify individuals who are infected with HIV through their HIV-positive contacts.
- Optimal index testing services depend largely on the ability to elicit sexual contacts from HIV-positive clients.
- Because some clients do not feel comfortable reporting their contacts, a low elicitation rate has been implicated in suboptimal testing of index contacts.
- The introduction of an elicitation box, in which an HIV-positive index can report sexual contacts on paper and insert in a box for a health care provider to contact at a later time, was expected to increase the elicitation rate of index contacts.
- Compared to the conventional elicitation model, the elicitation box model was found to be more likely to increase the rate of partners elicited, particularly for index clients with multiple sexual contacts.

**Key Implication:**

- HIV program managers should consider the introduction of the elicitation box model as an alternative strategy to increase partner elicitation.

## INTRODUCTION

HIV continues to be a significant global public health challenge, with approximately 38 million people living with HIV worldwide, two-thirds of whom reside in sub-Saharan Africa.^1^^,^^2^ Given that 15% of HIV-infected individuals in sub-Saharan Africa remain undiagnosed, improving access to HIV testing is crucial for epidemic control. WHO recommends a combination of facility and community-based approaches tailored to the specific needs of populations and geographies, such as safe and ethical index testing, social network strategy, HIV self-testing, provider-initiated testing and counseling, and targeted community testing.^3^^,^^4^

Index testing (also referred to as contact tracing, partner notification, or partner services) is an HIV testing modality that focuses on eliciting the sexual or needle-sharing partners and biological children of people living with HIV (PLHIV) and offering them HIV testing services.^5^ It is one of the most efficient and effective strategies for HIV case identification because over 80% of HIV infections occur in sexual and needle-sharing partners of PLHIV.^6^^–^^8^ A meta-analysis reported that positivity rates of individuals identified through index testing were high across all countries in sub-Saharan Africa, ranging from 21% in Zimbabwe to 51% in Nigeria.^9^^–^^13^

Index testing is one of the most efficient and effective strategies for HIV case identification.

Partner elicitation is the first step of the index testing process. At this stage, PLHIV, referred to as index clients, voluntarily provide contact details of their sexual and needle-sharing partners and biological children so they can be contacted, tested, and linked to prevention, care, and treatment services.^14^ Several studies have identified the fear of stigma and discrimination, especially among people with multiple past sexual partners, as a major barrier to accepting partner notification services.^15^^–^^18^ This leads to low elicitation rates. In Tanzania, Zambia, and Southwest Nigeria, studies documented an average elicitation ratio of 1:1.^19^^–^^21^ The studies found that clients with multiple sex partners were more likely to elicit numerous partners if there was a discreet method compared to directly disclosing to health care providers due to perceived self and social stigma.^19^^–^^21^

With funding from the U.S. Agency for International Development, FHI 360 piloted an elicitation box model as a discreet approach to contact elicitation in 4 of 21 local government areas (LGAs) in Akwa Ibom State, Nigeria, where the Strengthening Integrated Delivery of HIV/AIDS Services and the Meeting Targets and Maintaining Epidemic Control (SIDHAS/EpiC) projects were implemented. In 2018, Akwa Ibom State had the highest HIV prevalence and greatest unmet treatment need in Nigeria.^22^ This analysis assesses the elicitation and HIV- positivity rates of the elicitation box model compared to the conventional model in these pilot LGAs.

## METHODS

### Intervention Description

Index HIV testing in Nigeria is implemented in line with the national HIV treatment guidelines.^23^ Index clients who were newly diagnosed with HIV and index clients who visited the facility for antiretroviral therapy (ART) refill (either stable or unstable on ART) were counseled and asked to voluntarily and confidentially list and provide contact details of their sexual partners and offered the option of listing their sexual partners through direct elicitation (conventional model) or indirect elicitation (elicitation box model).

In the conventional method, index clients provided the details directly to the health care provider. Efforts were made to reach elicited partners for HIV testing through the index client (passive referral), the provider (provider-assisted referral), or both (dual and contracted referral approaches).^24^

**FIGURE 1:**
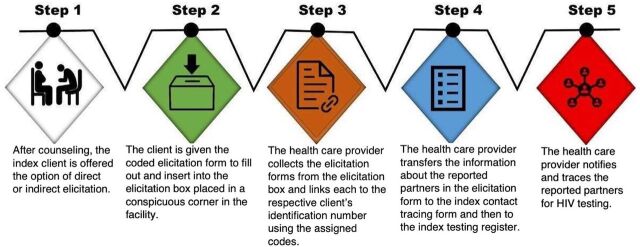
Service Flow of the Elicitation Box Model Implemented to Elicit Sexual Partners, HIV Treatment Health Facilities, Nigeria, March 2021–April 2022

In the elicitation box model (Figure 1), index clients wrote contact details of their sexual partners on an elicitation form pre-coded with the index client’s code and no other client identifiers, such as name or phone number, and inserted it into the elicitation box. The clients were aware that the forms were pre-coded. The health care provider then collected it from the elicitation box, matched the code with the client’s code, completed the index tracing form, and then entered information into the index register. The elicitation box, which was constructed of wood by project staff, was labeled “Elicitation Box” and placed in a conspicuous corner of the facility.

Akwa Ibom State, located in southern Nigeria, has a diverse population of 5.4 million, consisting of urban, metropolitan, riverine, agrarian, and coastal communities, including hard-to-reach areas. The SIDHAS/EpiC project provided HIV testing services in 21 of the 31 LGAs in Akwa Ibom, covering a total of 102 health facilities, including 1 tertiary facility, 20 secondary facilities, 15 private, for-profit facilities, and 66 primary health facilities. Index testing services are offered in all the supported facilities in Akwa Ibom State.

Specifically, this elicitation box model was implemented in facilities in 4 LGAs—Uyo, Oron, Ibesikpo, and Uruan. From March 1, 2021, to April 30, 2022, the intervention was implemented at 4 facilities, including 1 tertiary facility, 2 secondary facilities, and 1 primary health facility. The tertiary and 2 secondary-level facilities are located in urban areas, and the primary health facility is located in a semi-urban setting. The facilities had a high client load of at least 2,000 active clients on ART and a high HIV-testing volume. The settings had a high literacy rate among the population, including PLHIV.

### Data Collection

At the health facilities, service data are reported daily by trained data-entry clerks using paper-based forms, registers, and electronic medical records for program monitoring. Data were collated from the index register between March 2021 and April 2022 for the 4 health facilities on the following variables: sociodemographic characteristics of the index (age, sex, marital status, and type of index—defined as “newly identified positive,” “stable on ART” if already on ART and virally suppressed, or “unstable on ART”); mode of elicitation (direct, if the standard of care elicitation technique was used, or indirect, if elicitation box model was deployed); and characteristics of elicited contact, including age, sex, and HIV result.

### Outcomes Analyzed

The primary outcome of this analysis is the index partner elicitation ratio (the number of partners elicited divided by the number of HIV-index who accepted index testing services). Other outcomes measured include index testing acceptance (proportion of index HIV-positive clients who accepted index testing service); testing coverage (the proportion of partners HIV tested of partners elicited from HIV-index who accepted index testing services, including those with already known HIV-positive status); testing yield (the proportion of index partners identified as HIV positive from index partners HIV tested); and linkage rate (determined by the proportion of index partners identified HIV positive and linked to ART).

### Data Analysis

We conducted a retrospective analysis using routinely collected program data from service registers by counting the number of HIV-positive clients who received HIV index testing services at the selected facility level between March 1, 2021, and April 30, 2022. We conducted 4 separate analyses. First, we conducted summary statistics (frequency table), characterizing HIV- positive index clients offered index case testing across selected health facilities based on sex, age (included index clients aged 15 years and older), marital status, and client type (newly identified positive, stable, and unstable on ART), as well as characterize index partner contacts by elicitation type (conventional elicitation model vs. elicitation box model). Second, we created a flow chart cascade of events for index testing services across the selected HIV treatment health facilities. Third, we utilized chi-square (hypothesis testing) to determine if there was a difference in elicitation rate between the conventional elicitation model and elicitation box model.

A *P*-value of .05 or lower was considered statistically significant. Chi-square was used to carry out a comparative analysis based on choice of elicitation model by client characteristics across selected health facilities. Lastly, we compared index partner HIV testing coverage, testing yield, and linkage rate by elicitation approach (conventional elicitation model vs. elicitation box model) using chi-square test statistics. All statistical tests were conducted using SPSS 25.0.

### Ethical Approval

Ethical approval for the study was obtained from the Protection of Human Subjects Committee at FHI 360 (Project no. 1920318-1) and was determined to be non-human subject research.

## RESULTS

Of the 2,705 index clients offered index testing services between March 2021 and April 2022, the median age was 37 years, interquartile range: 31–43 years. The majority (66.3%) were female and married (54.8%). Most clients were stable on ART (n=2117, 79.3%) during the study period (Table 1).

**TABLE 1.** Characteristics of HIV-Positive Index Clients Offered Index Testing Services Across Selected Health Facilities, March 2021–April 2022

**N=2,705**	**No. (%)**
Sex	
Male	911 (33.7)
Female	1,794 (66.3)
Age, years	
15–24	194 (7.2)
25–49	2,189 (80.9)
50+	323 (11.9)
Marital status	
Single	908 (35.5)
Married	1,403 (54.8)
Separated/widowed	248 (9.7)
Type of client	
Newly identified positive	535 (20.0)
Stable on ART	2,117 (79.3)
Unstable on ART	19 (0.7)

Abbreviation: ART, antiretroviral therapy.

**FIGURE 2:**
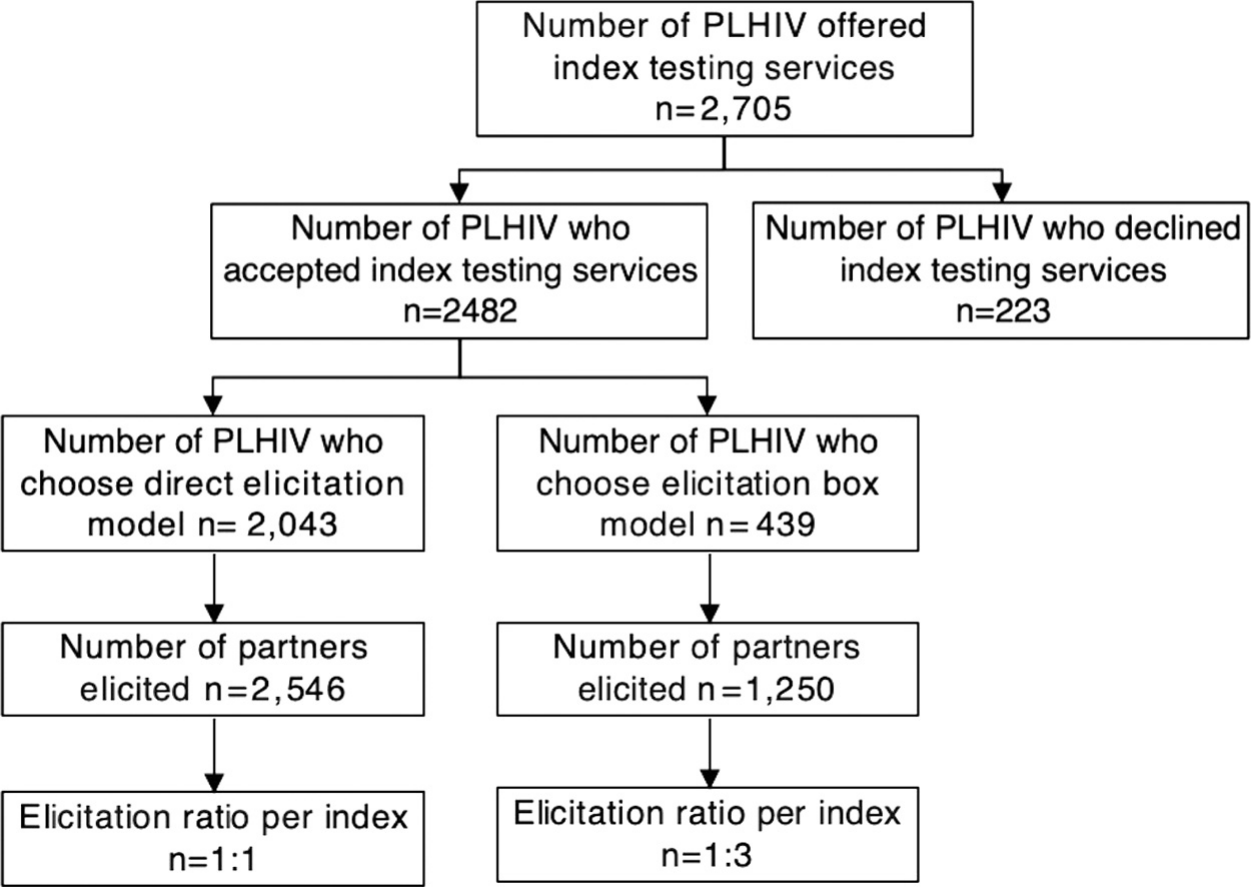
Flow Chart Showing Cascade of Event for Index Testing Services Across Selected HIV Treatment Health Facilities, Nigeria, March 2021–April 2022 Abbreviation: PLHIV, people living with HIV.

Of the 2,705 PLHIV offered index testing services, 2,482 (91.9%) accepted index testing, of which 82.3% (n=2,043) chose the direct elicitation model (Table 2), and 3,796 index partners were elicited. Of those index partners, 2,543 were from direct elicitation (index to partner ratio, 1 index: 1 contact) and 1,250 partners from indirect elicitation (index to partner elicitation ratio, 1 index: 3 contacts) (Figure 2).

**TABLE 2.** Comparative Analysis of Choice of Elicitation Model by Client Characteristics Across Selected Health Facilities, March 2021–April 2022

	**Choice of Elicitation, No. (%)**			
	**Conventional**	**Elicitation Box**	***P* Value**	**χ^2^**
Overall	2,043 (82.3)	439 (17.7)		
Sex				
Male	699 (81.4)	160 (18.6)	.37	0.79
Female	1,344 (82.8)	279 (17.2)		
Age (years)				
15–24	140 (75.7)	45 (24.3)	<.001	27.3
25–49	1,660 (81.5)	376 (18.5)		
50+	243 (93.1)	18 (6.9)		
Type of index				
Newly identified positive	400 (83.9)	77 (16.1)	.46	1.54
Stable on ART	1,602 (81.9)	354 (18.1)		
Unstable on ART	14 (77.8)	4 (22.2)		
Marital status				
Single	581 (70.9)	239 (29.1)	<.001	162.4
Married	1,259 (91.2)	121 (8.8)		
Separated/widowed	113 (72.9)	42 (27.1)		

Abbreviations: ART, antiretroviral therapy; χ^2^, chi-square test statistics.

The proportion of index clients who chose the elicitation box model was higher among index clients aged 15–24 years compared to other age groups (*P*<.001) and among those who were single compared to other marital status (*P*<.001) (Table 2).

Of the 3,796 index partners who were elicited from index testing services, the majority were male (n=2,449; 64.6%) and aged 25–49 years (n=3,199; 84.3%) (Table 3).

**TABLE 3.** Characteristics of Index Partner Contacts Listed by Elicitation Type

	**No. (%)**		
	**Total (N=3,789)**	**Conventional (n=2,543)**	**Elicitation Box (n=1,246)**
Sex^a^			
Male	2,449 (64.6)	1,646 (64.7)	803 (64.4)
Female	1,340 (35.4)	897 (35.3)	443 (35.6)
Age, years			
15–24	231 (6.1)	128 (5.0)	103 (8.3)
25–49	3,199 (84.3)	2,110 (82.9)	1,089 (87.3)
50+	363 (9.6)	307 (12.1)	56 (4.4)

a No sex documented for 7 elicited partners.

A total of 2,460 (64.8%) of the 3,796 elicited partners were found (testing coverage), tested for HIV, and received their results (67.1% in the conventional elicitation model compared to 60.1% in the elicitation box model). Of the 1,584 tested (testing yield), 202 (12.8%) were newly identified HIV-positive (conventional elicitation model: 12.9% [126/976]; elicitation box model: 12.5% [76/608]). Testing coverage was significantly higher in the conventional elicitation model when compared to the elicitation box model (*P*<.001). However, there was no significant difference in the testing yield (*P*=.81) and linkage rate using the conventional elicitation model compared to the elicitation box model (*P*=.13) (Table 4).

**TABLE 4.** HIV Testing Outcomes for Index Partner Contacts Listed by Elicitation Type

	**No. (%)**				
**Characteristics**	**Total (N=3,796)**	**Conventional Elicitation (n=2,546)**	**Elicitation Box (n=1,250)**	***P* Value**	**χ^2^**
HIV testing status					
Not tested	1,105 (29.1)	696 (27.3)	409 (32.7)		
Declined	231 (6.1)	141 (33.4)	90 (7.2)		
Negative	1,382 (36.4)	850 (33.4)	532 (42.6)		
Known HIV positive	876 (23.1)	733 (28.8)	143 (11.4)		
Newly identified HIV positive	202 (53.3)	126 (4.9)	76 (6.1)	.13	0.72
Testing coverage	2,460/3,796 (64.8)	1,709/2,546 (67.1)	751/1,250 (60.1)	<.001	18.24
Testing yield	202/1,584 (12.8)	126/976 (12.9)	76/608 (12.5)	.81	0.057
Linkage to ART	202/202 (100)	126/126 (100)	76/76 (100)	.13	0.72

Abbreviations: ART, antiretroviral therapy; χ^2^, chi-square test statistics.

## DISCUSSION

This analysis aims to evaluate the effectiveness of the elicitation box model in comparison with the conventional elicitation model in eliciting sexual partners. Findings from the analysis suggest that the use of the elicitation box model may be the model of choice for clients with multiple sexual partners; thus, including it as an option among high-risk populations is likely to generate more contacts than using only the conventional model.

Findings from the analysis suggest that the use of the elicitation box model may be the model of choice for clients with multiple sexual partners.

In this analysis, we present the yield of a novel and discreet alternative method to improve partner elicitation. Our goal was to reduce fear of self or social stigma in line with recommendations to encourage HIV-positive clients to fully participate in partner counseling and referral services.^25^ Index testing acceptance by HIV-positive clients in these 4 high-volume facilities was 91.9%. This finding aligns with a study that found a high acceptance level of 93% among the index clients offered index testing services in Zimbabwe and Tanzania^11^^,^^19^ and a 98% acceptance level in Lusaka.^20^ The acceptance rate may have been due to the introduction of the elicitation box, increasing client options for index testing. We found that the male clients had a higher acceptance rate (94.4%) compared to the female population. This is also consistent with studies conducted in Zimbabwe and Tanzania where partner elicitation is more promising in males compared to female index clients.^11^^,^^19^

Male clients reported 3 or more partners more frequently than female clients. This finding is consistent with a study that found that 42% of males elicited 2 or more partners compared to 25% of females.^8^ This could be because males are more likely than females to openly talk about the number of sex partners that they have, and females are less likely to name partners compared to males.^23^ Socially, females may fear cultural backlash, such as abandonment, violence, or other abuse, associated with partner elicitation in naming multiple partners.^26^ Additionally, there were more male partners (2,449) than females (1,347) reported by the index clients. This finding agrees with a study conducted in Cameroon that demonstrated that HIV partner services are an effective means of testing male partners.^27^

The choice of elicitation methods in this analysis was similar between males and females, with 18.6% of male clients and 17.2% of female clients choosing the elicitation box model. Index clients aged 15–24 years had the highest number of clients (24.3%) who chose the elicitation box. This can be associated with the confidentiality advantage that the indirect elicitation offers, coupled with the appreciable literacy level among this subpopulation.^28^ This model appears to be promising in the drive toward finding adolescents living with HIV who are an underserved population, as only 65% of adolescents living with HIV are on ART.^29^ Moreover, adolescents who may find it difficult to openly report sexual partners to adult health care workers need tailored approaches.^30^

A total of 1,089 partners (87.3%) aged 25–49 years were reported through the elicitation box model, which is the highest among all age groups. This suggests that this age group is likely to have had or currently have multiple sexual relationships that were discreetly reported.

Comparing conventional and elicitation box models based on the number of partners elicited through each model, we found that 2,043 index clients elicited 2,546 partners using the conventional elicitation model, resulting in an elicitation ratio of 1:1. Conversely, 439 index clients elicited 1,250 sexual partners through the elicitation box model, resulting in an elicitation ratio of 1:3. This finding, therefore, suggests that the indirect elicitation model may be effective in listing more sexual partners than the direct elicitation model.

The goal of index testing is to find PLHIV who are not on treatment and link them to ART services. The study showed that the positivity yield from partners elicited using the elicitation box (12.5%) and the conventional approach (12.9%) were comparable. This could likely be because the data of clients analyzed were mostly of those who were stable on ART and were virally suppressed, resulting in a low transmission rate. This finding concurs with a study that suggests that HIV-positive clients with a suppressed viral load of <1,000 copies/ml have almost zero risk of transmitting HIV to a sexual partner.^31^ It is important to note that the roll-out of pre-exposure prophylaxis to partners of index clients is likely to have influenced the positivity rate.^32^ Another possible reason for our low positivity rate is that the study setting is likely to be treatment saturated. This is consistent with the finding that the implementation of an ART surge in Akwa Ibom led to a 19% increase in viral suppression.^33^

This analysis appears to be the first to analyze routine program data of a novel and discreet program alternative to HIV partner elicitation. Key populations who suffer more stigma and labeling as a result of their sexual orientation may find this model very helpful in reporting their sexual contacts, as the confidentiality that this model provides will encourage them to report more sexual contacts, particularly in places where their sexual orientation is seen as a taboo. In settings where there is no audio-visual privacy for the client and health care provider to enable direct elicitation to take place, this model can close the gap. Also, the elicitation box model provides clients with an option for improving autonomy and agency.

### Challenges

A limitation of the elicitation box model is that it was restricted to clients who could read and write. Thus, the client’s literacy level could be a reason for the lower rate of choosing the elicitation box model over the conventional method. Further, when a client had illegible handwriting, it was difficult for the provider to identify and track the reported partners.

The cost of implementing the elicitation box was not stated for this intervention and may vary from country to country. However, countries wishing to implement the elicitation box model should budget for the construction of the elicitation box, printing of elicitation forms, and training of providers on implementation. Lastly, there was no chance to randomize clients to the elicitation model; because of this, it was not possible to understand what partners would have been elicited had the elicitation box model not been an option.

### Recommendation for Future Research

There is a need to increase the number of sites for implementing the elicitation box model for better coverage. It is also important to increase the number of index clients who obtained the index testing service for a robust analysis. Lastly, this model can be implemented in other geographical settings to either validate or invalidate the findings from this analysis. To draw a robust conclusion regarding the increase in testing numbers due to the introduction of the elicitation box, testing numbers need to be compared between facilities that have an elicitation box and those that do not.

### Limitations

Our study methods were retrospective; thus, there are a number of limitations. A comparative study was not done in these sites to compare testing coverage and yield between when the intervention was implemented and when it was not. In addition, there were limited data to show differences in people who chose the elicitation box compared to the conventional model, given that the elicitation box model was only implemented in 4 health facilities. Lastly, it is also not possible to know why people made the choice of the elicitation box.

## CONCLUSION

With the high elicitation ratio recorded from the elicitation box model, this analysis suggests that discreet models may increase multiple partner elicitation rates by reducing stigma. If implemented effectively, this approach may provide a possible alternative to the conventional elicitation method for improved case identification and achieving epidemic control.

## Notes

**Peer Reviewed**

**First Published Online:** September 24, 2024.
